# Current Knowledge and Recent Advances of Right Ventricular Molecular Biology and Metabolism from Congenital Heart Disease to Chronic Pulmonary Hypertension

**DOI:** 10.1155/2018/1981568

**Published:** 2018-01-17

**Authors:** Samantha Guimaron, Julien Guihaire, Myriam Amsallem, François Haddad, Elie Fadel, Olaf Mercier

**Affiliations:** ^1^Research and Innovation Unit, RHU BioArt Lung 2020, Marie Lannelongue Hospital, Paris-Sud University, Le Plessis-Robinson, France; ^2^Cardiac Surgery, Marie Lannelongue Hospital, Paris-Sud University, Le Plessis-Robinson, France; ^3^Division of Cardiovascular Medicine, Stanford Cardiovascular Institute, Stanford University School of Medicine, Stanford, CA, USA; ^4^Thoracic and Vascular Surgery and Heart and Lung Transplantation, Marie Lannelongue Hospital, Paris-Sud University, Le Plessis-Robinson, France

## Abstract

Studies about pulmonary hypertension and congenital heart diseases have introduced the concept of right ventricular remodeling leading these pathologies to a similar outcome: right ventricular failure. However right ventricular remodeling is also a physiological process that enables the normal fetal right ventricle to adapt at birth and gain its adult phenotype. The healthy mature right ventricle is exposed to low pulmonary vascular resistances and is compliant. However, in the setting of chronic pressure overload, as in pulmonary hypertension, or volume overload, as in congenital heart diseases, the right ventricle reverts back to a fetal phenotype to sustain its function. Mechanisms include angiogenic changes and concomitant increased metabolic activity to maintain energy production. Eventually, the remodeled right ventricle cannot resist the increased afterload, leading to right ventricular failure. After comparing the fetal and adult healthy right ventricles, we sought to review the main metabolic and cellular changes occurring in the setting of PH and CHD. Their association with RV function and potential impact on clinical practice will also be discussed.

## 1. Introduction

Recent emphasis on pulmonary hypertension (PH) has underscored the need for a better knowledge of the right ventricle mainly because studies have shown that left ventricular (LV) failure pathophysiology cannot be extrapolated to the right ventricle [1, 2]. Right ventricular (RV) physiology in health and disease has been gained thanks to studies in patients with pulmonary hypertension (PH) [3] and congenital heart disease (CHD) [4–6]. Even if PH is still defined as dramatic changes in pulmonary hemodynamics with mean pulmonary arterial pressure (mPAP) ≥ 25 mmHg at rest [7], right ventricular (RV) function is known as the major factor of functional capacity and prognosis in PH. PH is a global entity regrouping many subsets with different etiologies. Regardless of PH etiology, chronic pressure overload results in RV remodeling and adaptation and eventually leads to dysfunction and death in absence of lung transplantation. In a study investigating the immediate prognosis of RV failure in PH patients, the overall mortality was 14% but rose to 46% for patients requiring inotropic support and 49% for patients in the intensive care unit [8]. On the other hand, the mortality associated with LV failure requiring inotropic support is usually lower than 15% [9, 10]. Similarly, Humbert et al. reported survival rates of 85,7% at 1 year, 69,6% at 2 years, and 54,9% at 3 years, in patients with WHO group I PH admitted for RV failure [11].

Right heart failure in patients with PH is the result of insufficient blood delivery to the heart and/or increased systemic venous pressure secondary to elevated RV afterload represented by pulmonary arterial pressure or pulmonary vascular load [12]. The chronic RV pressure overload state results in myocardial remodeling, mainly characterized by a compensated hypertrophy. At the early stage, this adaptive right ventricle already presents with impaired bioenergetics, altered immunological response, and increased adrenergic response. This phenotype resembles the fetal RV phenotype; however, these adaptive mechanisms may lead to systolic dysfunction and cavities enlargement, representing the maladaptive right ventricle [13]. Clinical symptoms of peripheral edema, distended jugular veins, dyspnea, and syncope are consequences of increased filling pressures, diastolic dysfunction, and decreased cardiac output [3, 14, 15]. The transition from adaptive to maladaptive phenotype remains poorly understood and clinically unpredictable. Authors have been dividing this evolution into these 2 phenotypes, but there is a growing evidence for continuum between them: the adaptive right ventricle accumulates molecular and metabolic abnormalities until a point where it cannot overcome the persistent pressure overload and therefore becomes maladaptive.

Pulmonary arterial hypertension (PAH) associated with CHD belongs to group 1 of the WHO clinical classification of PH [7]. The evolution of this subset is characterized by dysfunction of the endothelial cells and hypertrophy and proliferation of the smooth muscle cells in the pulmonary circulation. Consequent obstruction of small arteries then occurs because of a narrowing of their diameter and plexiform lesions, the hallmark lesion of PAH. In this condition, pulmonary vascular resistances (PVR) progressively increase [16]. One of the difficulties to better appraise the natural history of RV failure is the variability of RV adaptation among patients exposed to chronic PH according to the etiology of PH. As an example, patients with PH related to untreated congenital cardiac defect or persistent ductus arteriosus may have a reversal of the left-to-right shunt, known as Eisenmenger syndrome. They tend to keep better RV function for a longer period and higher survival rate compared to patients with idiopathic PAH for a similar level of PVR and their survival is better [17–19]. Among the reasons to explain these different outcomes, the long-lasting fetal hypertrophied RV phenotype may prevent RV dilation and therefore its bowing towards the left ventricle. Second, persistent right to left shunts through septal defects enable tolerance of suprasystemic pulmonary hypertension [16].

The present review sought to summarize the current knowledge of RV metabolism and molecular physiology as studied in CHD and adult PH, in order to better understand RV failure associated with these diseases. After a brief overview of the fetal and adult right ventricle in health, we will review insights from experimental studies about pathophysiological evolution of RV remodeling in CHD and adult PH. Finally, we will develop the potential role of RV molecular biology and metabolism in the diagnosis, prognosis, and therapeutic approaches of RV dysfunction in PH. Inflammatory pattern of RV remodeling will be not described here as it has been recently well reviewed elsewhere [20, 21].

## 2. Right Ventricle in Health: From the Fetal Right Ventricle to the Adult Phenotype

### 2.1. Physiological Transition from the Fetal to the Adult Right Ventricle

The human heart originates from 3 main sources of cells: the first heart field, the second heart field and the neural crest cells. The first heart field gives the primitive left ventricle, a small part of the atria, and the atrioventricular canal myocardium resulting in the primary heart tube. The second heart field gives the complete right ventricle including the RV side of the ventricular septum and the RV outflow tract or pulmonary trunk. Finally, the neural crest cells participate in the constitution of the cardiac conduction system, as well as the RV outflow tract [22]. In utero, the right ventricle is not completely connected to the pulmonary circulation as it remains exposed to a high afterload and is not compliant. At this stage, the right ventricle plays the role of a systemic ventricle because of high PVR and low systemic vascular resistance in the placental circulation [22]. At birth, with the first breaths, fetal lung fluid is evacuated, partial O_2_ pressure increases, both creating an air-liquid interface, and ventilation occurs. These events result in increased shear stress in the pulmonary circulation leading to vasodilation secondary to the release of vasodilators, such as prostacyclin and nitric oxide, and decreased secretion of vasoconstrictors, such as endothelin-1. Since the placental circulation in utero is under high PVR and low systemic vascular resistance, clamping the umbilical cord at birth will therefore separate the newborn from the low resistance placental circulation, leading to a decrease in PVR and increase in systemic vascular tone. Concomitantly, patent ductus arteriosus progressively closes leading the right ventricle to eject only in the pulmonary arterial tree. As a result of these events occurring at birth, RV wall thickness progressively decreases and LV mass increases [22, 23]. This physiological transition occurs with important molecular, structural, and functional changes. Eventually, the right ventricle becomes more compliant and gains its adult phenotype, with normal relation and interdependence to the left ventricle.

### 2.2. Features of the Normal Fetal Right Ventricle

The fetal right ventricle is exposed to a low oxygen environment. Carbohydrates substrates are preferentially used to produce energy from the glycolytic pathway such as glucose, lactate, and pyruvate. The major signaling pathway expressed is the hypoxia inducible factor 1*α* (HIF1*α*) and vascular endothelial growth factor (VEGF) pathway, promoting angiogenesis. It is associated with upregulated glycolysis. Both of these mechanisms lead the fetal right ventricle to better tolerate hypoxia compared to the adult right ventricle [19, 22–24]. Moreover, because of patent ductus arteriosus, the fetal right ventricle is more sensitive to systemic vascular resistances than to PVR. Fisher et al. studied regional blood flow in fetal (*n* = 16), newborn (*n* = 12), and adult (*n* = 9) sheep, using radionuclide-labeled microsphere imaging [25]. He observed in all fetal lambs that regional blood flow was significantly higher in the right ventricular free wall compared to the left ventricular free wall, respectively, 213 ± 13 ml/min versus 162 ± 12 ml/min (*p* < 0.001). Similarly, the right side of the interventricular septum had a higher blood flow compared to the left side, respectively, 190 ± 13 ml/min versus 147 ± 12 ml/min (*p* < 0.05) [25]. Finally, microscopic features of the fetal right ventricle include small myocytes ranging from 5 to 7 *µ*m, a predominance of 70% of noncontractile mass represented of nuclei, cell membranes and mitochondria (30% of cardiomyocytes) [4], and disoriented sarcomeres with immature calcium homeostasis (Ca^2+^ pumps and transporters) and therefore immature contractility with genetic expression of *β*-myosin heavy chain (*β*-MHC) [24, 26, 27].

### 2.3. Features of the Normal Adult Right Ventricle

The adult right ventricle is exposed to a high oxygen environment with the adapted metabolic pathway including fatty acids oxidation, which produces more ATP than glycolysis [24]. Depending on injury or stress, there is an adaptation of metabolic substrates from fatty acids to glucose oxidation known as the Randle cycle. The HIF1*α*-angiogenesis signaling pathway and the glycolytic phenotype are no longer expressed in these conditions. In fact there are adult isoforms of enzymes such as pyruvate dehydrogenase kinase (PDK), especially the cardiac specific PDK4, which are responsible for glycolysis inhibition and promotion of glucose oxidation [21, 28]. In their study [25], Fisher et al. reported that, after birth, regional blood flow was higher in the left ventricular free wall compared to the right ventricular free wall, respectively, 204 ± 18 ml/min versus 140 ± 15 ml/min (*p* < 0.01). They explained this reversal in blood flow delivery by a change in oxygen requirements occurring after birth. In fact, because of the dramatic fall of PVR after birth, and therefore the decrease in right ventricular afterload, there is a major decrease in oxygen requirements for the right ventricle, with increased oxygen needs for the left ventricle being exposed to high systemic pressure [25]. Considering cellular features, myocytes are larger (from 15 to 25 *µ*m), sarcomeres are parallel, Ca^2+^ homeostasis is mature, and *β*-MHC gene is expressed [26], leading to a more efficient contractility [4, 27].  Table 1 summarizes the main differences between the normal fetal right ventricle and the normal adult right ventricle.

## 3. The Parallel between RV Remodeling in Pulmonary Hypertension and Congenital Heart Disease

### 3.1. Insights of Right Heart Failure in CHD

In conditions such as tetralogy of Fallot, surgically and congenitally corrected transposition of the great vessels, Ebstein anomaly, or late Fontan circulation, RV dysfunction is frequent because the right ventricle is chronically exposed to pressure overload. In the early phases, the right ventricle responds to increased wall stress with RV hypertrophy. It carries the role of a systemic ventricle characterized by coarse trabeculation, hypertrophied muscular band, and an abnormal septal tricuspid leaflet [29]. It increases its mitochondrial activity and carbohydrates use for energy production. Long-term evolution is characterized by substrates deprivation and energy loss. In addition, tricuspid regurgitation (congenitally corrected transposition of the great vessels and Ebstein anomaly), pulmonary regurgitation (Tetralogy of Fallot and RV dilation), and arrhythmias may occur [5]. Reasons for these evolution are multiple [29]. First of all, the right ventricle is characterized by a longitudinal contractile pattern compared to the circumferential pattern of the left ventricle (both radial and longitudinal). That makes the right ventricle unable to have a twisting and torsion component necessary to deal with high pressure overload. Second, RV tissue samples obtained in surgical CHD have shown decreased angiogenesis and marked fibrosis associated with arrhythmia, decreased RV ejection fraction, and increased RV wall stress [29, 30]. Third, studies showed that the hypertrophied right ventricle, because of its increased mass, has an impaired coronary flow and is therefore exposed to ischemia [19, 29, 30]. Finally, metabolic events include accumulation of mitochondrial reactive oxygen species (mROS) and of p53 protein responsible for HIF-1*α* signaling pathway inhibition and ventricular dilation as reported by Sano et al. in a mice model of transverse aortic constriction. At 14 days, maximum hypertrophy was reached, followed by loss of microvessels, ventricular dilation, and failure. When these mice were p53 knocked out, ventricular hypertrophy was sustained with high number of microvessels [31]. Another molecular feature has been described by Wu et al. on RV tissues from children with tetralogy of Fallot, hypoxia and hypertrophy (HH group), pulmonary stenosis, hypertrophy (H group), and small isolated ventricular septal defect compared to a control group. They reported that contractile dysfunction was linked to increased mROS associated with the decreased mRNA expression of a Ca^2+^-regulatory protein responsible for calcium homeostasis, as known as sarcoplasmic reticulum Ca^2+^-ATPase 2a (SERCA2a) [27].

### 3.2. Insights into Right Heart Failure in PH

**Figure 1:**
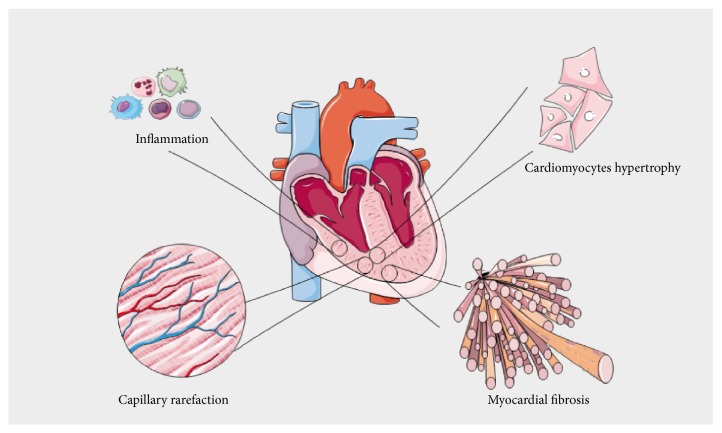
Main histological patterns of right ventricular remodeling in the setting of chronic pressure overload. Inflammation involving mononuclear cells and cardiomyocytes hypertrophy are observed at the early stage. Reduced capillary density and myocardial fibrosis are associated with right ventricular maladaptive phenotype.

Numerous experimental studies of RV remodeling in the setting of PH have been conducted in rodents including the fawn-hooded rat model [32] and then in the pulmonary arterial banding model, the hypoxia-induced PH [33], the angioproliferative PH, and the monocrotaline models [19]. The main histological features of RV tissular remodeling are summarized in Figure 1. Two phenotypes are usually described: “adaptive” versus “maladaptive” RV remodeling, or “compensated” RV hypertrophy (cRDVH) versus “decompensated” RV hypertrophy (dRDVH). That has been well described by Sutendra et al. especially by comparing baseline animals versus cRVH and dRVH in the same animals over time [19, 28, 34].

cRHV is characterized by numerous normal shaped hyperpolarized mitochondria, with low but continuous production of mROS, which does not allow p53 protein expression. This state is associated with high expression of HIF-1*α* pathway, increased levels of glucose transporter Glut-1, PDK4 enzyme, and therefore glucose uptake. These changes are responsible for a switch from a mitochondria-based glucose oxidation to a glycolytic status, as well as increased angiogenesis/VEGF expression [19]. Progressively accumulation of mROS leads to reduced number of mitochondria, sometimes clustered together, and usually with abnormal shapes and sizes [33, 35]. Finally, energy supply is insufficient with substrates deprivation and therefore dRHV. Activation of p53 protein and inhibition of HIF1*α* are key elements resulting in decreased angiogenesis and are associated with decreased PDK expression and glucose uptake leading to a reversed glycolytic shift. This last element was reflected by ^18^Fluorodeoxyglucose Positron Emission Tomography (^18^FDG-PET) showing increased glucose uptake in cRVH and reverse uptake in dRVH. In summary, a protective glycolytic shift appears to be associated with cRVH and reversed in dRVH because of excessive oxidative stress, substrates starvation, and mitochondrial loss of function [19, 34].

Concomitant of metabolic features, impaired angiogenesis is crucial in the pathology. Because of increased RV wall stress, myocardial oxygen consumption increases and therefore leads to loss of microvessels and reduced right coronary artery perfusion pressure (below 50 mmHg). This leads to an increased RV mass without compensatory angiogenesis and therefore results in ischemia [14, 36, 37]. Gómez et al. studied RV ischemia in patients with primary PH using stress technetium 99 m myocardial scintigraphy. RV ischemia was significantly correlated to increased RV end-diastolic pressure and increased right atrial pressure [14]. Bogaard et al. compared an isolated RV pressure overloaded rodent model (pulmonary arterial banding) versus a model with progressive pressure overload due to angioproliferative PH secondary to hypoxia and VEGF receptors blockage. In the context of angioproliferative PH, RV failure occurred with apoptosis, fibrosis, decreased VEGF gene and protein expressions, and decreased RV capillary density [36]. Finally, Tian et al. recently showed that RV ischemia causes mitochondrial-mediated fission that was responsible for diastolic dysfunction. When inhibiting mitochondrial fission, they showed a preserved RV function [38]. It is important to acknowledge that molecular and metabolic changes are a continuous process, starting as early as cRVH or “adaptive” RV remodeling. The precise role of the glycolytic shift, either protective or detrimental, remains a matter of debate and requires further investigations.  Table 2 summarizes characteristics of RV remodeling in CHD and PH.  Figure 3 depicts evolution of RV failure in CHD and PH.

Recent emphasis has been observed about the concept of right ventricular-pulmonary arterial (RV-PA) coupling as a relevant marker of cardiac performance and energetics for the right ventricle. It represents the maximal efficiency between stroke work and myocardial oxygen consumption. RV-PA coupling can be assessed using pressure-volume loops as the ratio between RV end-systolic elastance (Ees) and pulmonary arterial elastance (Ea) [2, 39]. Ventricular-arterial uncoupling is defined as Ees/Ea ratio below 1. At the early stage of PH, RV-PA coupling may be decreased, despite preserved RV function and increased contractility. When the elevated afterload is too high, RV stroke volume and RV ejection fraction decrease. Uncoupling therefore occurs, followed by RV dilation and failure [2, 40, 41]. Because pressure-volume loops assessment is invasive and time-consuming and may be dangerous for PH patients because of the need for transient but repeated occlusions of the inferior vena cava, this remains dedicated to experimental studies.

## 4. Clinical Perspectives

### 4.1. What Do RV Metabolic and Molecular Features Add to the Diagnosis of PH Related RV Dysfunction?

**Figure 2:**
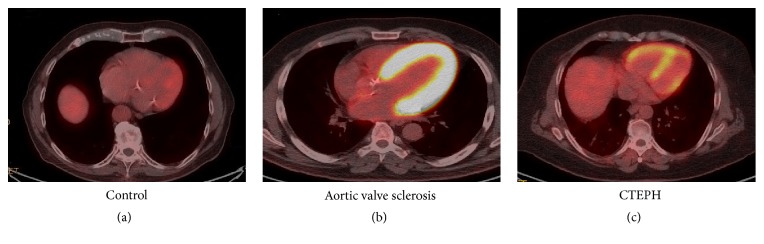
18-Fluorodeoxyglucose Positron Emission Tomography in a control healthy patient (a), in a patient with aortic valve sclerosis (b), and in a chronic thromboembolic pulmonary hypertension patient (CTEPH) (c). Images show 4 chambers views. Control imaging shows no right ventricular uptake, but presence of left ventricular uptake. Picture (b) shows increased glucose uptake localized on the left ventricular free wall and on the interventricular septum in a patient with marked hypertrophy of the left ventricle due to aortic sclerosis.

**Figure 3:**
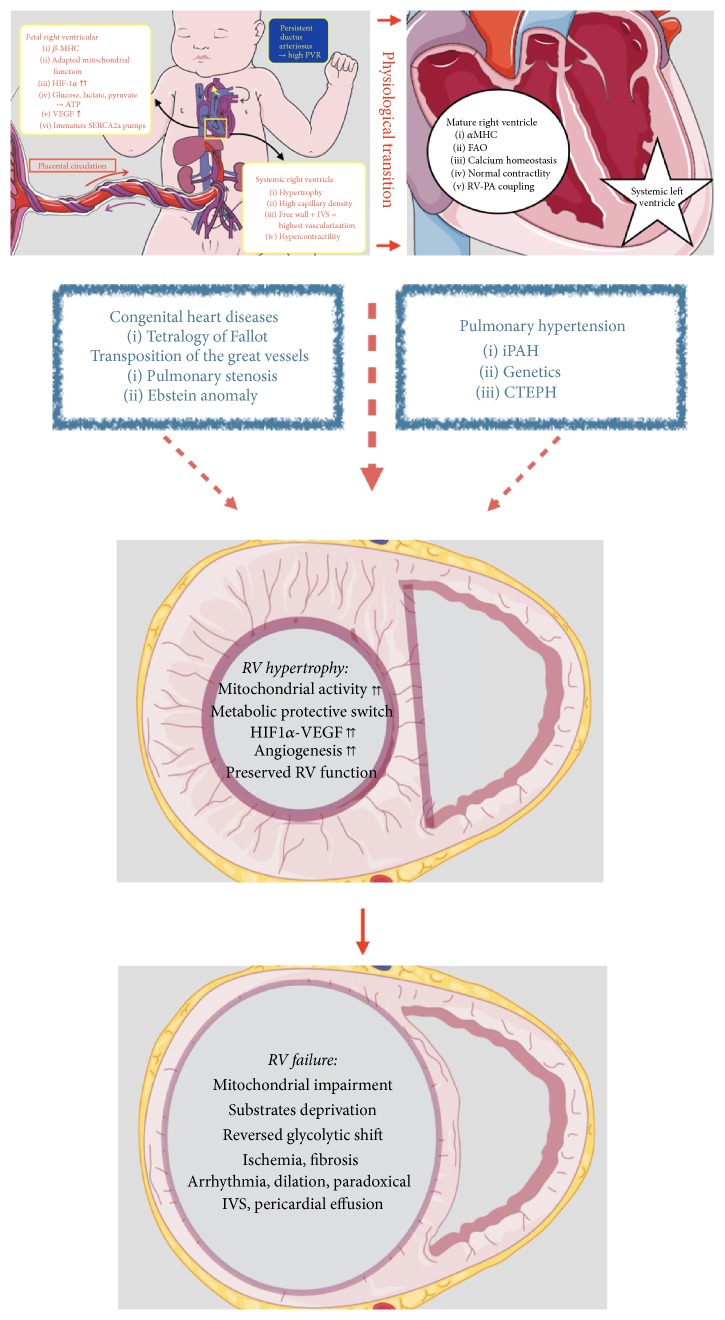
Right ventricular remodeling in congenital heart disease and pulmonary hypertension. *β*MHC: *β*-myosin heavy chain; HIF1*α*: hypoxia inducible factor 1*α*; VEGF: vascular endothelial growth factor; SERCA2a: sarcoplasmic/endoplasmic reticulum Ca2+ ATPase 2a; IVS: interventricular septum; *α*MHC: *α*-myosin heavy chain; FAO: fatty acids oxidation; RV-PA coupling: right ventricular-pulmonary arterial coupling; PVR: pulmonary vascular resistances; RV: right ventricular; iPAH: idiopathic pulmonary arterial hypertension; CTEPH: chronic thromboembolic pulmonary hypertension.

The glycolytic shift associated with RV remodeling is characterized by an upregulation of glucose uptake, shown by the increased uptake of ^18^FDG-PET [19, 37, 42, 43].  Figure 2 illustrates the hypermetabolism of hypertrophied RV observed in PH patients. Glucose uptake has been correlated with invasive PVR, mean pulmonary artery pressure, right atrial pressure, and RV wall stress [24, 44]. In addition, Lundgrin et al. showed the correlation between 18-FDG uptake and echocardiographic markers of systolic dysfunction (i.e., altered TAPSE, dilated RV, and RV fraction area change) and HIF-1*α* activation [45].

Brittain et al. hypothesized that alterations of FA metabolism were due to a decrease in FA oxidation. They focused on FA in blood samples, RV tissue samples, and their association with proton magnetic resonance spectroscopy. They observed increased levels of FA in the blood stream and in RV tissue samples. These findings were associated with cardiac steatosis and lipotoxicity on spectroscopy [46]. Studies investigating oxidative stress showed the key role of continuous mROS accumulation over time in the evolution of the pathology [19].

### 4.2. What Do RV Metabolic and Molecular Features Add to the Prognosis of PH Related RV Dysfunction?

The glycolytic shift observed in animal models of PH might be a marker for RV remodeling. Authors have studied PET imaging in PH patients in order to find a prognostic value of the observed metabolic changes. As previously mentioned, correlations between glucose uptake seen using 18FDG-PET imaging and hemodynamic have been reported, as well as association with echocardiographic findings and functional parameters such as the 6-minute walking test and NYHA status [47–49]. Moreover, there is evidence for the additional value of metabolic imaging for long-term follow-up of PH patients under treatment. Changes in FDG uptake over time seem to be related to varying expression of proangiogenic factors and different degrees of HIF-1*α* activation [45, 50]. Recently, Li et al. studied 45 patients with idiopathic PAH using PET imaging during fasting and glucose-loading conditions. They reported that increased RV to LV 18-FDG uptake ratio significantly predicted mortality [51]. These clinical findings therefore agree on the fact that PET imaging enables strong association between metabolism, mass, and RV function. Finally, in an ongoing clinical trial conducted by our team, we have been able to see significant correlations between decreased capillary density in human RV tissues and altered RV function (assessed by CMR and echocardiography) in the setting of chronic thromboembolic PH, as well as correlations with PET imaging.

### 4.3. What Do RV Metabolic and Molecular Features Add to Therapeutic Strategies of PH Related RV Dysfunction?

Phosphodiesterase-5, endothelin inhibitors, and prostaglandin D2 (PGD2) agonists have been shown to reduce RV pressure overload in patients with PH. However, their effects on the right ventricle are poorly explored. Authors hypothesized that myocardial substrates usage (such as glucose oxidation) can be modulated with pharmacologic inhibitors [24, 52]. Piao et al. studied the effect of PDK inhibitor, dichloroacetate, in two rat models of RV hypertrophy: monocrotaline-induced PH and pulmonary artery banding without PH. In the first model, dichloroacetate increased glucose oxidation and cardiac stroke work. Long-term use showed improved RV function. In RV compensated hypertrophy induced by pulmonary artery banding, the glycolytic shift could be reversed with dichloroacetate as well. These effects of dichloroacetate were greater in monocrotaline-induced RV hypertrophy associated with PH. Dichloroacetate might therefore correct vascular changes and RV remodeling [42]. These findings suggest that glycolysis is not detrimental for the overload right ventricle. Similarly, the same authors showed that long-term use of dichloroacetate inhibited FOXO1, a transcriptional regulatory factor of PDK. Consequent downregulation of PDK 4 (an isoform of PDK) restored glucose oxidation and improved bioenergetics and RV function [53].

The link between RV remodeling and fatty acid oxidation (the main energy source in healthy adult myocardium) still remains unclear. Fang et al. used partial inhibitors of FA oxidation such as ranolazine and trimetazidine in experimental pulmonary artery banding. They reported abnormal levels of FA oxidation with reduced RV function at baseline. Under ranolazine and trimetazidine treatment, they reported decreased FA oxidation and restored glucose oxidation resulting in increased cardiac output and improved exercise capacity [54]. Another metabolic-oriented therapy, as described earlier, which might be targeting mROS production with p53 protein inhibition might be an alternative [27]. Finally, beneficial effects might be possible by modulating mi-RNA expression in the heart in CHD or in the pulmonary arteries in PAH [6].

## 5. Conclusion

RV adaptation to pressure overload is a key determinant of survival in patients with PH and CHD. RV failure occurring in these conditions has similar features such as glycolytic shift and altered angiogenesis. A relationship between metabolic changes and RV function is strongly supported by recent experimental findings. Translational approaches of RV metabolism as well as noninvasive assessment of RV-PA coupling are needed to better discriminate the RV phenotype in the setting of chronic pressure overload. Pharmacological support to modulate RV energetics and to restore RV capillary density might be promising approaches to improve the condition of PH patients.

## Figures and Tables

**Table 1:** Main characteristics of healthy phenotypes of fetal and adult right ventricles.

Characteristics	Fetal phenotype	Adult phenotype
*Environment*		
Oxygen environment	Low	High
Main blood circulation	Placental circulation	Systemic circulation
Ductus arteriosus	Opened	Closed
PVR	High	Low
Main vascularized heart regions	Right ventricular free wall, Right side of the IVS	Left ventricular free wall, Left side of the IVS
Systemic ventricle	Right ventricle	Left ventricle
*Genetics*		
Gene pattern expression	*β*-MHC	*α*-MHC
*Metabolic features*		
Mitochondrial function	Normal/adapted	Normal/adapted
mROS production	Adapted to heart activity	Adapted to heart activity
Energetic substrates	Carbohydrates	Fatty acids
Hypoxia-induced factors		
(i) HIF1*α* (ii) VEGF	Expressed	Not expressed
Ca^2+^ homeostasis	Immature	Mature
*Cellular features*		
Myocytes diameter	5–7 *µ*m	15–25 *µ*m
Myocytes/nonmyocytes ratio	30%	70%
Sarcomeres	Disoriented	Parallel
Capillary density	Preserved	Preserved
Fibrosis	Absent	Absent

PVR: pulmonary vascular resistance; MHC: myosin heavy chain; mROS: mitochondrial reactive oxygen species; HIF-1*α*: hypoxia inducible factor 1 alpha; VEGF: vascular endothelial growth factor; Ca^2+^: calcium.

**Table 2:** Common features of functional and dysfunctional remodeled right ventricles in congenital heart disease and pulmonary hypertension.

Characteristics	Functional remodeled right ventricle	Dysfunctional remodeled right ventricle

*Morphology*		
Chambers size	Normal	Dilated (i.e., RV/LV > 0,6)
Free wall thickness	Thick (>5 mm)	Thin
IVS motion	Normal	End-diastolic bowing in the left ventricle
Pericardial effusion	Absent or minimal	Moderate to important
CHD common features	Coarse trabeculation Hypertrophied and muscular moderator band Abnormal tricuspid septal leaflet insertion (mitral valve proximity)	
*Function*		
RVEF	Preserved	Decreased
Contractility	Hypercontractility	Decreased
Cardiac index	Preserved	Decreased Bad prognosis < 2 l/min/m^2^
RV-arterial coupling	Preserved	Uncoupling
Rhythm	Mostly preserved	Arrhythmias
CHD common features	Tricupid and pulmonary regurgitations prior to dilation	
*Metabolic features*		
Mitochondria	Adapted sizes and shapes	Small, abnormal shapes, clustered
Mitochondrial function	Increased	Decreased
mROS production	Continuous and Low	High accumulation
Signaling pathway	Down-regulation of p53 Up-regulation of HIF1*α*-VEGF pathway	Up-regulation of p53 Inhibition of HIF1*α*-VEGF pathway
Energetic substrates	Carbohydrates > fatty acids High use of PDK4, Glut1 = glycolytic shift	Total substrates deprivation Energy starvation = reversed glycolytic shift
*Cellular and Tissular features*		
Myocytes	Hypertrophied	?
Capillary density	Increased	Rarefaction
Ischemia	Present With role of CHD-associated coronary malformations	Present
Fibrosis	Absent	Present

IVS: inter entricular septum; RVEF: right ventricular ejection fraction; CHD: congenital heart disease; RV-arterial coupling: right ventricular arterial coupling; mROS: mitochondrial reactive oxygen species; p53: p53 protein; HIF-1*α*: hypoxia inducible factor 1 alpha; VEGF: vascular endothelial growth factor; PDK4: pyruvate dehydrogenase kinase 4; Glut1: glucose transporter 1.
